# Biosynthesis of gold nanoparticles using leaf extract of *Dittrichia viscosa* and in vivo assessment of its anti-diabetic efficacy

**DOI:** 10.1007/s13346-022-01163-0

**Published:** 2022-04-30

**Authors:** Sanaa Ayyoub, Bahaa Al-Trad, Alaa A. A. Aljabali, Walhan Alshaer, Mazhar Al Zoubi, Sahar Omari, Diaa Fayyad, Murtaza M. Tambuwala

**Affiliations:** 1 Department of Biological Sciences, Faculty of Science, Yarmouk University, Irbid, 21163 Jordan; 2 Department of Pharmaceutics and Pharmaceutical Technology, Faculty of Pharmacy, Yarmouk University, Irbid, 21163 Jordan; 3 Cell Therapy Center, University of Jordan, Amman, Jordan; 4 Department of Basic Medical Sciences, Faculty of Medicine, Yarmouk University, Irbid, 21163 Jordan; 5 School of Pharmacy & Pharmaceutical Sciences, Ulster University, Coleraine, BT52 1SA Northern Ireland UK

**Keywords:** Diabetes mellitus, Gold nanoparticles, Nanomedicine, Hepatic gluconeogenesis, Type 2 diabetes, *Dittrichia viscosa*, PEPCK activity

## Abstract

Several studies have reported the anti-diabetic effect of biologically synthesized gold nanoparticles (AuNPs). This study was designed to investigate the in vivo anti-diabetic activity of AuNPs synthesized using the leaf extract of *Dittrichia viscosa* in a high-fat diet (HFD)/streptozotocin (STZ)-induced diabetes in rats. AuNPs were synthesized using the leaf extract of *D. viscosa*, and the synthesized AuNPs were characterized by UV–visible spectrophotometer, dynamic light scattering (DLS), zeta potential, and transmission electron microscopy (TEM). To study the anti-hyperglycemic effect of the AuNPs formed using *D. viscosa* extract, adult male Sprague–Dawley rats were divided into three groups (6–8 rats/group) as follows: control group, a diabetic group without treatment, and a diabetic group treated intraperitoneally with a daily injection of AuNPs at a dose of 2.5 mg/kg for 21 days. Diabetes was induced by maintaining the rats on HFD for 2 weeks, followed by a single intraperitoneal injection of 45 mg/kg of STZ. Serum and liver samples were collected at the end of the treatment period and used to measure glucose levels and hepatic gene expression and activity of phosphoenolpyruvate carboxykinase (PEPCK), the rate-limiting enzyme in the liver gluconeogenic pathway. The AuNPs formed using *D. viscosa* extract were mainly spherical with a size range between 20 and 50 nm with good stability and dispersity, as indicated by the zeta potential and DLS measurements. Treatment with AuNP significantly lowered the blood glucose level, the gene expression, and the activity of hepatic PEPCK in comparison to the diabetic untreated group (*P* < 0.05). This study suggests that AuNPs synthesized using *D. viscosa* leaf extract can alleviate hyperglycemia in HFD/STZ-induced diabetes in rats, which could be through the reduction of hepatic gluconeogenesis by inhibiting the expression and activity of the hepatic PEPCK gene.

**Graphical abstract:**

Schematic illustration of the biosynthesis of AuNPs showing their distinctive morphology under the EM. The generated particles were injected into animals and serum glucose levels were reported in addition to the PEPCK expression and activity

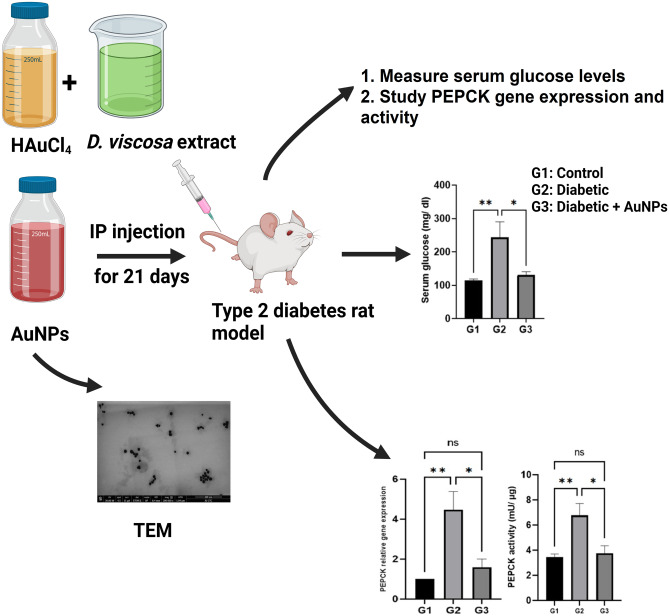

## Introduction

Diabetes mellitus (DM) is a chronic metabolic disorder with the main characteristic of elevated blood glucose levels, either due to insufficient or lack of insulin secretion, resistance to its action, or both [1, 2]. The most common classifications of DM by etiology and clinical presentation include type 1 diabetes mellitus (T1DM), type 2 diabetes mellitus (T2DM), and gestational diabetes [2, 3]. T1DM occurs primarily due to autoimmune destruction of insulin-producing β cells in the islets of the pancreas, which accounts for 5–10% of cases, while T2DM accounts for ~ 90% of all cases of diabetes [2]. T2DM is characterized by insulin resistance, pancreatic β cells dysfunction, and insulin deficiency [3]. In addition, the hepatic gluconeogenic pathway, which synthesizes glucose from non-carbohydrate precursors, is abnormally activated during type 1 and 2 DM, causing hyperglycemia even in the fasting state [4]. Therefore, suppressing the hepatic gluconeogenic pathway is one of the suggested options to manage elevated blood glucose levels, and any compound that can inhibit this pathway can be of therapeutic importance for the management of diabetes.

New trends are currently emerging for the management of DM, where nanotechnology has gained significant interest because of the advantages it provides and the success it showed in several areas of medicine. Nanomedicine refers to the use of materials in which at least one of its dimensions is less than 100 nm [5] for various medical applications, including disease treatment, diagnostic purposes, or understanding of disease mechanisms.

Among the recent findings in the field is discovering the anti-diabetic effect of metallic nanoparticles, including gold nanoparticles (AuNPs) [6]. Initial studies on AuNPs have shown promising results in this field in lowering blood glucose and lipid levels and its protective effect against complications associated with diabetes [7–9]. Several studies have confirmed the anti-diabetic effect of AuNPs using extracts from different plant sources for their synthesis [10–13].

*Dittrichia viscosa*, also known as false yellow head [14] and initially classified in the *Inula* genus [15], is commonly used in traditional medicine as an anti-inflammatory and anti-microbial agent [16]. Other studies have also demonstrated its anti-cancer and anti-diabetic effects [17, 18]. To our knowledge, no study has demonstrated the biological synthesis of AuNPs using the extract of *D. viscosa*; neither their anti-diabetic effect has not been evaluated. Additionally, the effect of AuNPs on the expression and activity of the rate-limiting enzyme in hepatic gluconeogenesis has not been studied. This study addressed these issues by evaluating the in vivo anti-diabetic activity of AuNPs synthesized using the leaf extract of *D. viscosa* in HFD/STZ-induced diabetes in rats.

## Material and methods

### Chemicals and materials

Tetrachloroauric acid and streptozotocin (Sigma-Aldrich, USA), glucose GOD-PAP (Fortress diagnostics, UK), total RNA purification kit (Jena Bioscience, Germany), PrimeScript™ RT master mix (Takara, Japan), BlasTaq™ 2X qPCR MasterMix (Applied Biological Materials, Canada), protein quantification kit BCA assay, (Abbkine, China), phosphoenolpyruvate carboxykinase activity assay kit (MyBioSource, USA).

### Synthesis and characterization of the gold nanoparticles

Biosynthesis of AuNPs was conducted using dried leaf extract of *D. viscosa*, collected in October 2020 from North of Jordan. Leaves of *D. viscosa* were dried in an oven at 37 °C overnight and were stored at room temperature away from light.

Leaf extract was prepared by adding 1 g of the dried plant to 100 ml of boiling water with continuous stirring for 15 min and then it was kept cooling at room temperature and filtered using Whatman No. 1 filter paper. The extract was then added to 1 mM aqueous HAuCl_4_ solution at a volume ratio of 1:5 followed by measurement of absorbance at a resolution of 2 nm and wavelength range between 240 and 800 nm at 4-h intervals in a quartz cuvette with a path length of 1 cm. After the reaction had completed, the solution was centrifuged at 10,000 rpm for 20 min and the pellet was weighed and resuspended in distilled water.

Surface charge measurement and the formed NPs hydrodynamic diameter were determined using Zetasizer Nano ZS90 (Malvern Panalytical, UK), where 100 µl of the solution was suspended in 900 µl of distilled water. Measurements were taken under the following conditions: 25 °C, 1.33 dispersant refractive index, and 0.8872 cP viscosity. The shape of the formed nanoparticles was determined by TEM using a Titan FEI microscope, where the sample was first dispersed in water, deposited on a carbon grid, and allowed to dry prior to imaging.

### Animal studies

All experimental animal procedures were carried out according to the National Institutes of Health (NIH) guide for the care and use of laboratory animals and approved by the animal ethics committee at Yarmouk University (ACUC/2021/10).

### Induction of type 2 diabetes in rats and treatment

Adult male Sprague–Dawley rats, 3 months of age and weighing between 150 and 200 g, were obtained and kept at the animal in Yarmouk University. Rats were divided into three groups (*n* = 6–8/group): control (non-diabetic), diabetic without any treatment, and diabetic treated with a daily intraperitoneal injection of AuNPs at a dose of 2.5 mg/kg for 21 days [9]. Type 2 diabetes was induced by maintaining the rats on HFD for 2 weeks; the diet consisted of egg yolk and butter mixed with feed. This was followed by a single intraperitoneal injection of freshly prepared STZ (45 mg/kg) dissolved in 50 mM sodium citrate buffer (pH 4.5). Three days following the injection, blood glucose levels were measured in fasting rats using a glucometer (GlucoLab, Infopia, Korea); rats with blood glucose levels higher than 150 mg/dl were considered diabetic.

### Blood glucose measurement

Blood samples were collected in the fed state by heart puncture and were placed in plain tubes for serum collection and kept undisturbed for 15 min, followed by centrifugation at 10,000 rpm for 10 min. According to the manufacturer’s protocol, glucose levels of serum samples were determined using the GOD-PAP colorimetric method (Fortress diagnostics, UK).

### Measurement of PEPCK enzyme activity

Liver tissue homogenate was prepared by homogenizing the liver sample with ice-cold phosphate buffer saline (PBS) at a ratio of 1 g tissue:9 ml PBS on ice. The homogenates were then centrifuged at 5000 g and 4 °C for 5 min to get the supernatant. The protein concentration was determined for all samples with a protein quantification kit using the bicinchoninic acid (BCA) assay method according to the manufacturer’s protocol (Abbkine, Inc. China).

The homogenates were diluted with PEPCK assay buffer (1:2) for the measurement of the enzyme’s activity. According to the supplier’s protocol, a colorimetric detection method was used (MyBioSource, Inc., USA). Final absorbance was measured in kinetic mode at 570 nm and 37 °C, for 55 min at 5-min intervals. Two-time points were selected for all the samples to calculate PEPCK activity.

### Real-time PCR

The quantitative gene expression of hepatic PEPCK was determined using RT-PCR. First, total RNA was extracted from liver samples using a spin column-based method based on the instructions of the supplier’s protocol (Jena Bioscience). RNA concentration was quantified by measuring absorbance at 260 nm using µDrop plate, Multiskan GO, and SkanIt software (Thermo Scientific, USA). The RNA was then reverse transcribed into cDNA using a commercial kit (Takara, Japan). The reaction mixture was placed in a thermocycler at 37 °C for 15 min, followed by inactivation of reverse transcriptase at 85 °C for 5 s.

BlasTaq™ 2X qPCR MasterMix was used for the RT-PCR step. The reaction components included the cDNA sample, forward and reverse primers, BlasTaq™ 2X qPCR MasterMix, and nuclease-free water with a final volume of 20 µl. The samples were placed in the RT-PCR thermocycler LineGene 9600 (Bioer Technology Co., China), and the reaction conditions were as shown in Table 1. The cycling parameters were as follows: 95 °C for 3 min and 45 cycles of 95 °C for 3 s and 60 °C for 30 s. Relative mRNA concentrations were normalized to the housekeeping gene GAPDH. The fold changes in mRNA expression were determined using the 2^−ΔΔCT^ method [19]. The sequence of the primers used is shown in Table 1.

**Table 1:** Sequences of primers used for quantitative real-time RT-PCR

**Gene**	**Forward primer (5′-3′)**	**Reverse primer (5′-3′)**	**Reference**
**GAPDH**	ATGGTGAAGGTCGGTGTG	GAACTTGCCGTGGGTAGA	[35]
**PEPCK-C**	CCCAGGAAGTGAGGAAGTTTGT	GGAGCCGTCGCAGATGTG	[36]

*PEPCK-C* phosphoenolpyruvate carboxykinase, cytosolic, *GAPDH* glyceraldehyde 3-phosphate dehydrogenase

### Statistical analysis

One-way analysis of variance (ANOVA) test was performed for data analysis using SPSS software version 23 (SPSS Inc., Chicago, IL), and graphs were created using GraphPad Prism version 9.0.0. Statistical significance was considered when the *P*-value was less than 0.05.

## Results

### AuNPs characterization

**Fig. 1:**
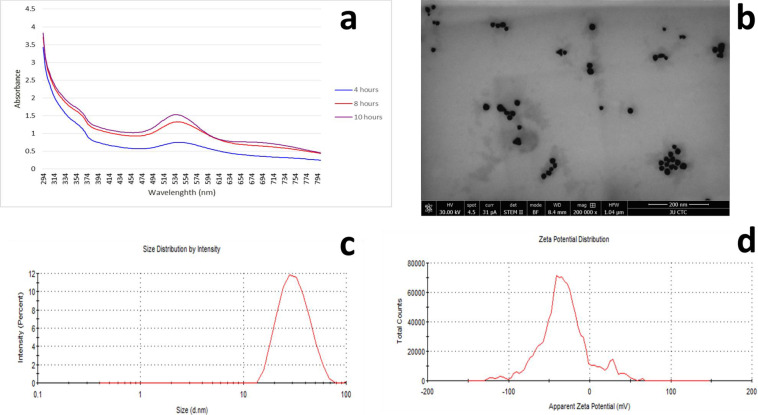
Results of the AuNPs characterization. **a** UV–vis absorption spectra over time of AuNPs synthesized using *D. viscosa* leaf extract. **b** Unstained TEM image of the formed AuNPs. **c** AuNPs’ size distribution as determined by DLS. **d** The zeta potential (*ζ*) distribution of the generated AuNPs

Figure 1 shows the characterization results of the formed AuNPs, as follows: panel (a) shows the UV–visible spectrophotometer absorbance spectra over time and panel (b) shows the TEM image of the formed nanoparticles with approximate size between 20 and 50 nm in diameter. DLS results showed that the average hydrodynamic diameter was following the results obtained by the TEM, as shown in Fig. 1c. Zeta potential measurements indicated that the surface charge of NPs was − 29.3 mV, as shown in Fig. 1d.

The UV–vis spectra showed peak absorbance at 536 nm and no significant changes in peak absorbance were observed after 10 h of the reaction. According to the TEM results, over 95% of the generated NPs were spherical with very few triangular-shaped NPs. This was confirmed by the TEM images of the nanoparticles using ImageJ software for particle counting and distribution. The analysis revealed that approximately 95% of the imaged particles were spherical and monodisperse.

### Rats’ weight and blood glucose levels

**Fig. 2:**
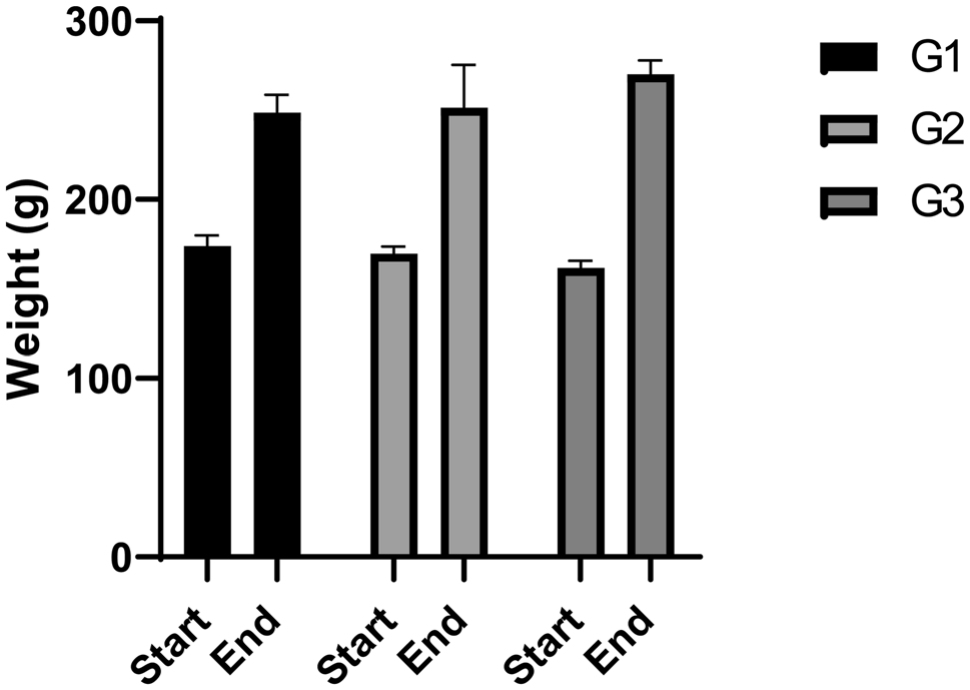
Weight of rats (g) at the beginning and end of the experiment (G1, control; G2, diabetic; G3, diabetic treated with AuNPs)

**Fig. 3:**
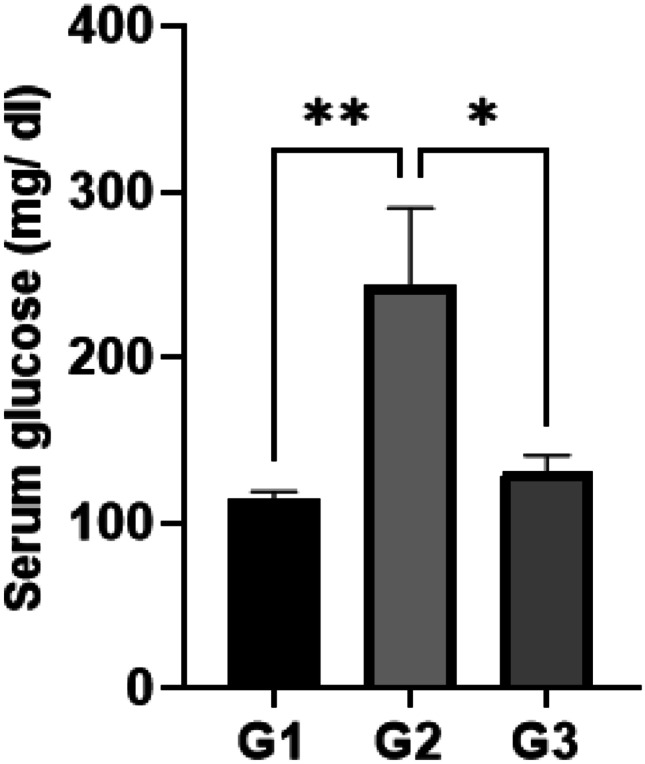
Blood glucose level (mg/dl) expressed as mean ± SE (G1, control; G2, diabetic; G3, diabetic treated with AuNPs; *P = 0.0193, **P = 0.0034)

Initial and final body weight was not different among groups (Fig. 2). After 21 days, diabetic rats showed significant increases in blood glucose compared to the non-diabetic group (*P* < 0.01; Fig. 3). Treatment with the biologically synthesized AuNPs significantly decreased blood glucose level compared to the diabetic group (*P* < 0.05; Fig. 3).

### Effect of AuNPs on the expression of the hepatic PEPCK and its activity

**Fig. 4:**
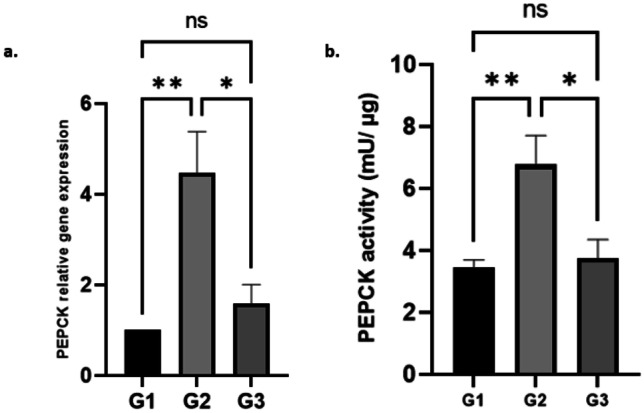
**a** The relative gene expression of PEPCK among experimental groups (G1, control; G2, diabetic; G3, diabetic treated with AuNPs; ns, non-significant; *P = 0.0138, **P = 0.033). **b** Hepatic PEPCK enzyme activity (G1, control; G2, diabetic; G3, diabetic treated with AuNPs; ns, non-significant; *P = 0.0156, **P = 0.0078)

Results of PEPCK gene expression shown in Fig. 4a revealed that the PEPCK mRNA expression level was significantly increased in the diabetic group compared to the non-diabetic group, and treatment with the AuNPs significantly decreased the PEPCK mRNA expression levels with no significant differences between the treated groups and the control group. Also, the hepatic PEPCK enzyme activity was significantly lowered when treated with the AuNPs compared to the diabetic group (*P* < 0.02), as shown in Fig. 4b.

## Discussion

Diabetes mellitus is a chronic metabolic disorder characterized by persistent hyperglycemia. T2DM occurs because of a combined impairment in insulin secretion (β cell failure) and insulin action (insulin resistance) [2, 3, 20]. HFD/STZ-induced diabetes in rats is a common model of T2DM. The HFD contributes to hyperglycemia, hyperinsulinemia, and insulin resistance caused by obesity, while STZ destroys the pancreatic β cells, which are all a characteristic of T2DM [21]. In searching for new, effective, and safe anti-diabetic drugs, AuNPs received tremendous attention as they can be potentially used to manage diabetes [6]. Therefore, this study was designed to investigate the in vivo anti-diabetic activity of AuNPs synthesized using the leaf extract of *D. viscosa* in HFD/STZ-induced diabetes in rats.

In the current study, a biological approach was used to synthesize the AuNPs, where the leaf extract of *D. viscosa* was used as a reducing agent. Plant extracts contain several compounds and functional groups that can reduce and stabilize agents in the synthesizing of metallic NPs [22]. This includes flavonoids, terpenoids, hydroxyl, carboxyl, and others that act through different mechanisms to reduce gold ions into AuNPs.

AuNPs synthesized using plant extract usually have better stability over chemically synthesized AuNPs. In this study, the surface charge was − 29.3 mV in the aqueous media, indicating good stability of the formed NPs due to repletion between particles and a low possibility of aggregation [23]. The NPs mainly were spherical with a size of between 20 and 50 nm.

Several studies have reported the anti-diabetic effect of AuNPs in different diabetic animal models, where the effect has been attributed mainly to the anti-oxidant properties exerted by these particles. AuNPs have managed to restore the activity of several anti-oxidant enzymes that are known to be diminished in diabetes and maintain the redox balance [6]. It has also been reported that AuNPs can scavenge free radicals and are ten times more powerful than vitamin E and five times more powerful than vitamin C as an anti-oxidant [24].

It was clear that AuNPs managed to significantly lower the serum glucose levels to normal compared to the diabetic group. Several mechanisms have been suggested for the hypoglycemic effect exerted by AuNPs. In one study, AuNPs synthesized using guavanoic acid had an inhibitory effect on protein tyrosine phosphatase 1B (PTP 1B) [25], which is involved in the insulin signaling pathway and is considered a promising target for T2DM management [26]. This eventually leads to enhancing glucose uptake in the muscle and adipose tissue. Other studies showed that AuNPs have an inhibitory effect on some digestive enzymes, including α-amylase and α-glucosidase [27, 28], which reduces glucose release. Another study suggested that AuNPs interact with the cysteine residues present in an enzyme involved in regulating oxidative stress known as thioredoxin, thus, preventing an inhibitory protein from binding to it and enhancing their anti-oxidant activity [27].

Also, plant extracts are known to act as reducing and capping agents in synthesizing metallic nanoparticles. Therefore, the anti-diabetic agents present in the plant extract, including flavonoids, tannins, terpenoids, alkaloids, and others [28], can be concentrated on the surface of the formed NPs, thus improving their anti-diabetic effect. Thus, the effect may be attributed to both the AuNPs, and the hypoglycemic agents present in the plant’s extract. Also, polyphenols, abundant in *D. viscosa* extract [29], are known to exhibit anti-diabetic effects acting through different mechanisms [30], including inhibiting glucose absorption in the intestine through inhibiting glucosidase and amylase activity [31], and increasing glucagon-like peptide 1 (GLP1) secretion and its half-life by inhibiting dipeptidyl peptidase-4 (DPP4) and by increasing insulin secretion and insulin sensitivity [32].

Fasting hyperglycemia in T2DM has been shown to be a function of increased hepatic gluconeogenesis [33]. In the current study, the relative mRNA expression and the activity of a key gluconeogenic enzyme, PEPCK, were significantly lowered in the group treated with the AuNPs compared to the diabetic group. The hypoglycemic effect of AuNPs in type 2 diabetes may be due to the suppression of expression of PEPCK. This effect could be due to the ability of AuNP to regenerate the pancreatic cells, therefore, enhancing insulin secretion [34], as it is known that insulin acts as a repressor for PEPCK expression.

## Conclusion

This study suggests that AuNPs synthesized using the leaf extract of *D. viscosa* can alleviate hyperglycemia in HFD/STZ-induced diabetes in rats that could be through reducing hepatic gluconeogenesis by inhibiting hepatic PEPCK mRNA expression levels and its protein activity.

## Data Availability

All data can be available.
